# Comprehensive sexuality education in primary education: analyzing the impact of an educational intervention on knowledge, perceptions, and communication—a quasi-experimental study

**DOI:** 10.3389/fpsyg.2026.1815472

**Published:** 2026-07-21

**Authors:** Mónica Raquel Pereira Afonso, Elena Arroyo-Bello, Raquel Fernandez-Cezar, Victoria Lopezosa Villajos, Daniel Díaz Díaz, Maria Angélica de Almeida Peres, Sonsoles Hernández-Iglesias, Sagrario Gomez Cantarino

**Affiliations:** 1 Faculty of Physiotherapy and Nursing, University of Castilla-La Mancha, Toledo, Spain; 2 Fundación Jiménez Díaz School of Nursing - Autonomous University of Madrid, Madrid, Spain; 3 Health Research Institute-Fundación Jiménez Díaz University Hospital, Universidad Autónoma de Madrid (IIS-FJD, UAM), Madrid, Spain; 4 Critical Perspective Research Group, Department of Mathematics, Faculty of Education of Toledo, University of Castilla-La Mancha, Toledo, Spain; 5 Anna Nery School of Nursing, Federal University of Rio de Janeiro, Rio de Janeiro, Brazil; 6 Faculty of Health Sciences, Francisco de Vitoria University, Madrid, Spain

**Keywords:** adolescence, intervention, risk behavior, sexual education, youth

## Abstract

**Introduction:**

Comprehensive sexuality education is fundamental for the healthy development of children and adolescents, as it promotes essential knowledge, values, and skills for responsible emotional and sexual lives. Early and integrated implementation within the school curriculum contributes to the prevention of risks such as sexually transmitted infections (STIs), unintended pregnancies, and exposure to pornography. This study aimed to explore short-term changes following a school-based sexual health education intervention among primary school students.

**Methods:**

A quantitative pre-post approach was employed using a quasi-experimental design with a single intervention group. Participants were assessed through pre- and post-tests using non probabilistic sampling. The educational intervention addressed topics including sexuality, puberty, consent, anatomical knowledge, and critical understanding of sexual content.

**Results:**

The intervention was associated with increased understanding of sexuality, puberty, and consent among students. Improvements were observed in anatomical knowledge as well as in students’ comfort and openness when discussing sexual health topics. Levels of exposure to sexual content were identified, highlighting the relevance of early and age-appropriate education. Prior to the intervention, students reported limited knowledge about sexuality and puberty and relied primarily on family as their main source of information.

**Discussion:**

Educational interventions may help normalize discussions about sexuality and improve communication among primary school students. However, insufficient responses to abuse situations and increasing exposure to pornography emphasize the need for comprehensive, critical, and age-appropriate sexuality education from early educational stages.

## 1 Introduction

Education aims to equip individuals for effective societal participation, representing one of the most critical tools for both individual and collective development. Its role is to transform and improve personal circumstances while actively shaping social interactions (Borazon and Chuang, 2023). Over time, education and health have evolved in tandem, converging into the concept of health education, which is defined as a series of planned and systematic educational interventions designed to enhance population health literacy (Estrela et al., 2025). This encompasses not only increasing knowledge regarding health determinants and healthy practices but also fostering personal skills and competencies that support the adoption of health-enhancing behaviors (Akyol et al., 2023). Within this framework, health education serves as a key instrument for promoting wellbeing across healthcare, social, and educational domains.

Sex education constitutes a vital component of this synergy, addressing the physical, emotional, and social dimensions of sexuality throughout the lifespan (Kim et al., 2023; Vidal and Montes, 2025). The World Health Organization (WHO) recognizes Comprehensive Sexuality Education (CSE) as an essential educational need based on a holistic perspective of childhood and adolescence (UNESCO and WHO, 2023). Consequently, it must be integrated transversally and systematically into curricula through interdisciplinary actions (Myat et al., 2024). Furthermore, it is a right endorsed by the United Nations Educational, Scientific and Cultural Organization (UNESCO), which advocates for initiating this instruction at an early stage, adapting it progressively and coherently to students’ developmental phases (UNESCO et al., 2025). This is particularly critical in contexts where students often do not transition from primary to secondary education, highlighting the necessity of providing essential information during the earliest stages of schooling (Nguyen et al., 2025).

Scientific evidence demonstrates that inclusive and transversal sex education programs yield benefits that extend far beyond risk prevention. These include promoting respect for diversity, reducing bullying and homophobic attitudes, fostering a deeper understanding of gender norms, and enhancing the valuation of equity and social justice, thereby positively impacting the school climate and socio-emotional development (Goldfarb and Lieberman, 2021). Furthermore, CSE directly contributes to the sustainable development goals (SDGs) of the 2030 Agenda, most notably SDG 3 (Good Health and Wellbeing), SDG 4 (Quality Education), and SDG 5 (Gender Equality) (United Nations, n.d.).

For CSE to be effective, its implementation must be timely and developmentally relevant; for instance, addressing pubertal changes shortly before their biological onset (UNESCO and WHO, 2023). Beyond objective data, early exposure to these contents shapes the self-perception of sexual knowledge (the subjective assessment of what individuals believe they know). This cognitive variable represents a relevant factor for self-confidence when asking questions, seeking information, and preventing behaviors associated with sexual risk (Silva et al., 2024).

In Spain, affective-sexual education is constitutionally supported under the Organic Law on the General Organization of the Educational System (LOGSE) (Jefatura del Estado, 1990), and the current educational framework (LOMLOE) reinforces its importance by explicitly incorporating contents on diversity, identity, and sexual orientation (Jefatura del Estado, 2020). Nonetheless, the transmission of these values confronts complex cultural and religious patterns that act as barriers within families and communities (Jefatura del Estado, 2020; Jiménez-Ríos et al., 2023). These taboos often silence open dialogue, leaving many adolescents, particularly in rural environments, without clear guidance (Jiménez-Ríos et al., 2023; Markham et al., 2014).

Adolescence represents a critical period characterized by physical, psychological, and social transformations that impact identity formation (Herzig van Wees et al., 2021), a stage wherein interpersonal relationships are actively explored and autonomy is developed (Inchley et al., 2020). The absence of formal and scientific education during this phase allows fundamental concepts to become distorted through unreliable sources (Mavhandu-Mudzusi and Mhongo, 2021). In fact, pornography consumption among adolescents occurs at increasingly earlier ages (Sanjuan-Meza et al., 2019). Because sexuality is present throughout the lifespan (Sladden et al., 2021), there is an imperative need to equip students with tools to manage their health (Borazon and Chuang, 2023; UNESCO and WHO, 2023); nevertheless, previous studies indicate that knowledge regarding sexuality remains insufficient among children and adolescents (Myat et al., 2024; Nguyen et al., 2025; Jefatura del Estado, 2020).

Therefore, this study focuses on analyzing the short-term changes associated with an educational intervention on the level of sexual knowledge among primary education students.

The objective of the present research is to analyze the short-term changes associated with an intervention designed to enhance knowledge regarding sexuality among primary education students, while also evaluating its influence on attitudes, behavioral intentions, and self-reported exposure to sexual health content. This general objective is broken down into the following hypotheses:

*H1*: Primary education students demonstrate significant deficiencies in their knowledge of sex education, particularly regarding sexuality and sexual health.

*H2*: The implementation of educational interventions in sexual health positively affects the improvement of students’ knowledge and attitudes within this field.

## 2 Materials and methods

### 2.1 Study design

A quantitative quasi-experimental single-group pre-post design was employed via non probabilistic sampling. Data collection took place between October 2024 and February 2025.

### 2.2 Participants

The study was conducted in three public primary schools in the Toledo area (Castilla-La Mancha, Spain) during the 2024–2025 academic year. The sample consisted of students enrolled in the final 2 years of primary education (5th and 6th grades), ranging in age from 10 to 12 years.

The inclusion criteria were as follows: (1) enrollment in a public primary school and (2) basic reading and writing skills sufficient to understand and independently complete the questionnaire. Exclusion criteria included: (1) students attending private or semi-private schools, (2) those absent from class on the day of the intervention, and (3) those who had not provided signed informed consent from their families.

In the pretest phase, 80 students completed the questionnaire; in the posttest phase, 69 students completed the questionnaire. Because the questionnaires were anonymous and unpaired, the analytic sample size varied by time point and by item due to occasional non-responses.

### 2.3 Ethical considerations

The study was approved by the Social Research Ethics Committee of the University of Castilla–La Mancha (CAU-661803-V4Z4) and was conducted in accordance with the Declaration of Helsinki.

Participation was voluntary, and students’ right to withdraw from the questionnaire at any time was guaranteed. Informed consent was obtained through the completion of a form by the families, which included information on data confidentiality and the anonymous nature of the questionnaire. Age-appropriate information was provided to the students before administration, and they were informed that they could skip any questions.

### 2.4 Intervention

A structured, age-adapted sexual health education workshop was developed for 5th- and 6th-grade students. The intervention was conducted in a single 2-h session delivered in the classroom during school hours. It covered several key topics related to sexual health. First, the concept of sexuality was explored, including its meaning and its relationship with gender, identity, and sexual orientation. An explanation was then given for the changes that occur during childhood and adolescence, including physical, psychological, and emotional transformations. Next, a playful activity focusing on sexual consent was carried out, emphasizing its importance and presenting different scenarios that the students had to classify as consensual or nonconsensual. Finally, pornography was addressed, with special emphasis on the associated risks, such as sexual violence, and the potential development of addictive behaviors related to its consumption.

Thus, a structured educational intervention was developed that integrated multimedia resources such as videos and illustrations, along with well-founded and precise responses, adapted to the demands and cognitive level of the students.

The workshop used interactive and participatory strategies (guided discussion, scenario based classification activity, and audiovisual support) to facilitate a safe, non-stigmatizing environment for questions and dialog.

### 2.5 Instrument

A questionnaire on sexual knowledge—particularly focusing on puberty, consent, and pornography—was specifically developed for the purposes of this study (Table 1). The questionnaire was developed by the research team on the basis of a targeted review of the literature and prior instruments used in educational settings. The design was informed by previous instruments used by other authors (Sim-Sim et al., 2022; Gradellini et al., 2022; Mecugni et al., 2021; Davis, 1992; Soto-Fernández et al., 2024).

**Table 1:** Sexuality survey for primary education students.

Item	Response
Dimension 1: Foundational knowledge of sexuality	
Do you know what the word “sexuality” means?	Yes No I’m not sure
Who can you ask if you have questions about sexuality? (Check all the options you think are correct)	Family Friends Teacher Healthcare professional
How do you feel when you talk about sexuality?	Comfortable Uncomfortable Normal I have never talked about this before
Do you know what puberty is?	Yes No
Dimension 2: Perceptions and attitudes towards sexual consent	
Why do you think it is important to learn about sexuality? (Mark all options you think are correct)	a) To know my body b) To understand the changes in puberty c) To learn about respect and boundaries d) To avoid false information from the internet e) I do not know why it is important
What would you do if someone touched you in a way that makes you feel uncomfortable?	a) I would tell them to stop and walk away b) I would keep it a secret c) I would tell a trusted adult d) I do not know what I would do
Dimension 3: Exposure to pornography	
Have you ever seen any sexual videos or photographs?	Yes No If you answered YES, where did you see them? ___________________________

*Source*: Authors’ own elaboration.

The content validity of the sexuality survey designed for primary school students was assessed through an expert panel, following the Davis technique (Roldán Restrepo et al., 2021) for instrument validation. The panel consisted of seven specialists, selected on the basis of their academic background and professional experience in areas directly related to the construct under study: three experts in sexuality, one psychologist, one pediatric nurse, one linguistics expert, and one primary school teacher. This multidisciplinary composition ensured a comprehensive evaluation of the instrument, integrating theoretical, educational, health-related, and linguistic perspectives.

The evaluation process was conducted via an online questionnaire that included all the survey items. Each expert independently assessed the relevance of each item using a four point ordinal scale, in accordance with the criteria proposed by Davis:

1. The item is not appropriate for measuring the construct and should be eliminated.
2. Needs major revision: the item is partially related to the construct but requires substantial modifications.
3. Relevant but needs minor revision: the item is appropriate, although small wording or content adjustments are needed.
4. This is highly relevant: the item is clear, appropriate, and accurately represents the construct, requiring no modifications.

For quantitative analysis, the Item-Level Content Validity Index (I-CVI) was calculated by considering ratings of 3 or 4 as indicative of content validity. The I-CVI was obtained by dividing the number of experts assigning these ratings by the total number of experts. In addition, the Scale-Level Content Validity Index (S-CVI/Ave) was calculated as the average of the I-CVI values across all items of the instrument.

Following the initial independent evaluation, a consensus process was conducted. Expert comments and quantitative results were reviewed collectively, and discrepancies or suggestions for improvement were discussed until agreement was reached regarding the final wording and suitability of the items. This consensus phase ensured both conceptual coherence and linguistic appropriateness of the instrument for the target population. Furthermore, qualitative feedback provided by the experts during the consensus process led to minor wording revisions aimed at enhancing clarity, comprehensibility, and developmental appropriateness for primary school students.

Questionnaires were administered anonymously and were not pseudonymized to minimize any risk of identification in a minor population and to promote a safe environment that could facilitate honest responses. Therefore, individual responses could not be linked across the pre- and posttests, and comparisons were conducted between two unpaired samples drawn from the same school setting.

The questionnaire was structured into three dimensions, comprising a total of eight multiple-choice items. These dimensions were (1) basic knowledge of sexuality (four items); (2) perceptions and attitudes toward sexual consent (two items); and (3) exposure to pornography (one item), as shown in Table 1.

The primary outcomes were item-level changes in self-reported understanding of sexuality and puberty, as well as changes in consent-related items; the secondary outcomes included comfort in discussing sexuality and self-reported exposure to sexual content.

Given the heterogeneous item formats (single-choice categorical items, multiple-response items, and one open-ended question), analyses were conducted at the item level rather than as a summed scale score.

### 2.6 Procedure

School administrators were contacted by the research team and provided with detailed information about the objectives and procedures. Families were informed through the schools and provided written informed consent.

Participation was voluntary, and students’ right to withdraw from the questionnaire at any time was guaranteed. Informed consent was obtained through the completion of a form by the families, which included information on data confidentiality and the anonymous nature of the questionnaire. Intervention sessions were organized in coordination with the teaching staff at each school and were conducted according to a schedule agreed upon from October 2024 to February 2025. With respect to the intervention’s development, the pretest questionnaire was provided to each school to be administered at least 2 weeks before the re-searchers’ visit. The posttest was distributed 1 week after the sessions. The participating schools’ teachers returned all questionnaires to the researchers.

The questionnaires were completed anonymously in the classroom. Because questionnaires were not pseudonymized, individual matching between pre- and posttests was not possible, and analyses compared unpaired samples.

A structured sexual health educational intervention was implemented for primary school students. All the sessions were delivered by three nursing professionals with training and experience in health education and pediatric sexual health. Of these, two nurses conducted the sessions, while a third supervised adherence to the intervention guide, ensuring consistency in delivery and minimizing facilitator related variability.

Prior to the sessions, a comprehensive intervention guide was developed and reviewed by a panel of experts in pediatric sexual education, psychology, pedagogy, and linguistics. This standardized guide included all content, activities, materials, and timing for each component of the intervention and was followed rigorously in every classroom to ensure intervention fidelity.

The intervention was structured into six sequential components designed to promote progressive learning tailored to students’ developmental levels. At the start of each session, ground rules were established to create a safe, respectful, and emotionally neutral environment for discussing sensitive topics. Additionally, a “question box” was provided, allowing students to submit questions anonymously, which were addressed at the end of the session, reinforcing comprehension and participation.

During the sessions, active participation was encouraged, allowing students to intervene at any time to ask questions or clarify concepts, as well as to engage in interactive activities and educational games. These included exercises focused on identifying personal boundaries and consent, in which students evaluated various everyday scenarios to determine whether consent was present, promoting respect for oneself and others and the development of decision-making skills.

All the content was carefully adapted to the students’ cognitive and linguistic levels, including sensitive topics such as pubertal physiological changes, sexuality, digital safety, and exposure to pornography, addressed in an age-appropriate and preventive manner. Activities were supported by visual materials, practical scenarios, and participatory exercises to ensure understanding and meaningful learning.

Each session concluded with a wrap-up and consolidation activity, reinforcing key learning points and addressing the questions submitted in the question box. The standardized design of the intervention, the consistent involvement of the same professionals, and the supervision of adherence to the guide throughout the sessions contributed to strengthening intervention fidelity and the internal validity of the study.

The intervention consisted of a single 2-h classroom workshop delivered during school hours by a healthcare professional member of the research team in coordination with teaching staff. Table 2 summarizes the intervention components, learning objectives, activities, materials, and corresponding competencies used to support replication.

**Table 2:** Intervention description.

Component	Learning objective	Activities/delivery	Dose	Materials	Competency link
1. Ground rules	Create a safe, neutral space for sensitive topics.	*“The Question Box”*: Set up an anonymous box for questions to be answered at the end.	10 min	Slips of paper, box, slides.	Psychological safety & comfort.
2. Defining sexuality	Destigmatize the term and explain its scope.	*Concept mapping*: Brief lecture on how sexuality includes feelings, identity, and biology.	20 min	PowerPoint, visual aids.	Holistic understanding of self.
3. Puberty	Normalize physiological and emotional changes.	*Myth vs. Fact*: Use “True/False” paddles or movement-based voting (stand up/sit down).	25 min	Diagram of hormonal changes, anatomical image of the reproductive system; slides.	Biological literacy.
4. Consent	Define physical boundaries and “The Power of No.”	*Scenario sorting*: Categorize behaviors into “Safe,” “Unsafe,” or “Ask First.”	35 min	Scenario cards, boundary maps.	Self-protection & agency.
5. Digital safety	Build critical thinking regarding online content.	*The “Trusted Adult” circle*: Identifying three people they can talk to if they see something confusing.	20 min	Digital vignettes, “Help-seeking” worksheet.	Media literacy & safety.
6. Wrap-up	Consolidate learning and provide a safety net.	*Exit ticket*: One thing they learned + answering anonymous questions from the box.	10 min	Resource handouts/stickers.	Resource navigation.

*Source*: Authors’ own elaboration.

### 2.7 Statistical analysis

Statistical analyses were conducted using Stata software version 18.0. A comparative analysis of responses obtained in the pre- and post-intervention phases was performed at the item level. Because questionnaires were completed anonymously and responses could not be linked across time points, pre- and post-intervention data were analyzed as unmatched samples. Descriptive statistics included absolute and relative frequencies (percentages) for each response category in both phases. For inferential analysis, Pearson’s chi-square test and, when required due to small cell counts, Fisher’s exact test were used to compare response distributions between phases. Effect size was quantified for each item using Cramér’s *V*. Statistical significance was set at *p* < 0.05 (two-tailed).

## 3 Results

The Sexuality Survey for Primary Education students was analyzed, with 80 responses in the pre-intervention phase (pretest) and 69 responses after the intervention (posttest).

### 3.1 Dimension 1: critical knowledge of human sexuality: from prior understanding to con-structed learning

Dimension 1 summarizes students’ self-reported foundational understanding of sexuality and puberty, their feelings when discussing sexuality, and their perceived sources of support for sexuality-related questions. Table 3 presents the distribution of responses at pretest and posttest for Dimension 1 items, along with group-level comparisons between unpaired samples (Fisher’s exact) and effect sizes (Cramér’s *V*).

**Table 3:** Dimension 1 outcomes by study phase (pre vs. post).

Item	Response category	Pre *n*/*N* (%)	Post *n*/*N* (%)	*χ*^2^ (df)	*p* (Fisher, 2-tailed)
P1. Do you know what the word “sexuality” means?	No	12/80 (15.00)	1/69 (1.45)	34.89 (2)	<0.001* V Cramer 0.484
	Yes	35/80 (43.75)	62/69 (89.86)		
	I’m not sure/I have doubts	33/80 (41.25)	6/69 (8.70)		
P2. Do you know what puberty is?	No	23/75 (30.67)	11/69 (15.94)	4.32 (1)	0.049* V Cramer 0.173
	Yes	52/75 (69.33)	58/69 (84.06)		
P3. How do you feel when you talk about sexuality?	Comfortable	10/79 (12.66)	18/67 (26.87)	7.43 (3)	0.062 V. Cramer 0.226
	Uncomfortable	19/79 (24.05)	14/67 (20.90)		
	Normal	41/79 (51.90)	33/67 (49.25)		
	I have never talked about this before	9/79 (11.39)	2/67 (2.99)		
	Yes	35/77 (45.45)	38/68 (55.88)		

*Source*: Authors’ own elaboration. *Bold values indicate statistically significant results (*p* < 0.05). p values were calculated using Fisher’s exact two-tailed test. Cramer’s V is reported as a measure of effect size for the association between categorical variables.

Table 3 summarizes the students’ responses by study phase. Understanding of the term ‘sexuality’ differed across phases, with a greater percentage answering ‘Yes’ at post-test (89.86%) than at pre-test (43.75%) (Fisher’s exact *p* < 0.001). Knowledge of ‘puberty’ also differed across phases (84.06% vs. 69.33% answering ‘Yes’; Fisher’s exact *p* = 0.049). With respect to feelings when talking about sexuality, the distributions showed a nonsignificant trend (Fisher’s exact *p* = 0.062), with fewer students reporting that they had never talked about the topic at post-test.

As shown in Table 3, the proportion of students reporting that they understood the term ‘sexuality’ was higher at post-test than at pre-test (Fisher’s exact *p* < 0.001; Cramer’s *V* = 0.484). Self-reported knowledge of ‘puberty’ also differed across phases (*p* = 0.049; *V* = 0.173). Feelings when talking about sexuality showed a nonsignificant trend (*p* = 0.062; *V* = 0.226), with fewer students reporting that they had never discussed the topic.

**Figure 1:**
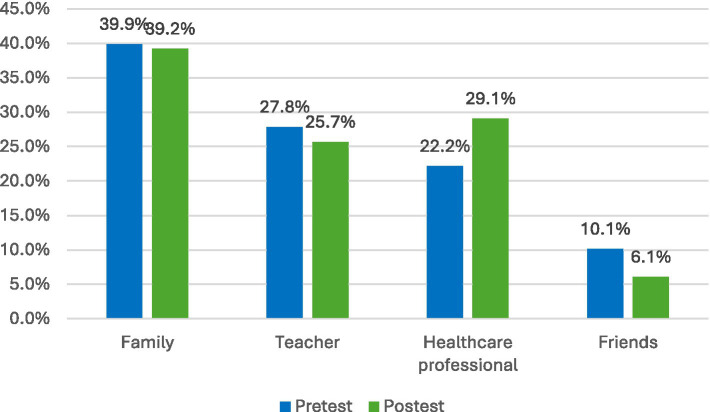
Who can you ask if you have questions about sexuality?

Regarding the question ‘Whom can you turn to if you have questions about sexuality?’ Figure 1 illustrates the responses reported by participants during the pre- and post-intervention assessments. In both phases, two participants provided no response.

### 3.2 Dimension 2: recognition and validation of consent in affective-sexual situations

**Figure 2:**
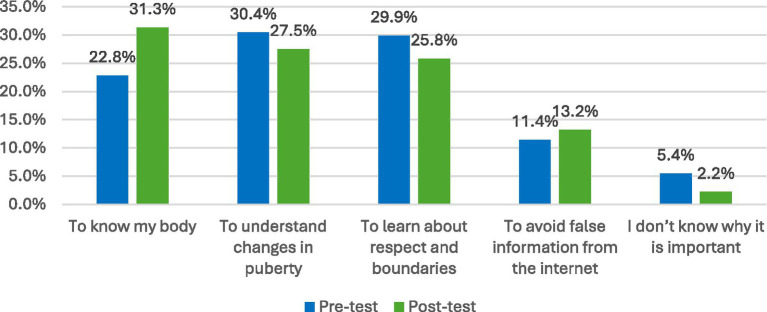
Reasons to learn about sexuality (multiple-response item).

Dimension 2 summarizes students’ consent-related perceptions, including reasons to learn about sexuality, recognition of private body parts, and intended responses to uncomfortable touching. Figure 2 shows the proportion of students selecting each reason for learning about sexuality (multiple responses allowed) at the pretest and posttest. All the students answered the question.

Furthermore, students’ motivations for considering sexual education important were explored through a multiple-response question. In the pretest phase, the most frequently cited reasons were “understanding puberty changes” (*n* = 56) and “knowing limits and respect toward others” (*n* = 55). However, in the posttest phase, a redistribution of priorities was observed. The most selected option was “knowing my own body” (*n* = 57), followed by “understanding puberty changes” (*n* = 50) and “learning about respect and boundaries” (*n* = 47). This shift suggests an evolution toward a more personalized and reflective perception of sexuality, in which bodily self-knowledge is positioned as the foundation for a healthy and conscious experience of this dimension.

**Figure 3:**
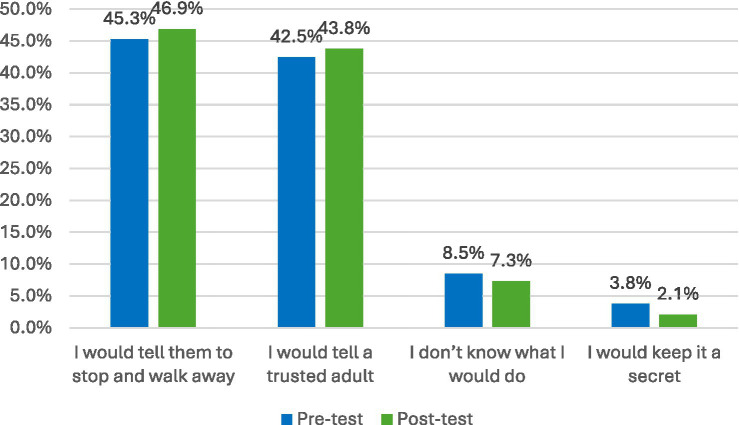
Intended response to uncomfortable touching (single-choice).

We next examine students’ intended behavioral responses to uncomfortable touching, assessed through the item “What would you do if someone touched you in a way that made you feel uncomfortable?”. Thirteen students did not answer this question at pretest, compared with three students who did not answer in the post-intervention phase. Response distributions are reported by study phase (pre- and post-intervention) in Figure 3.

### 3.3 Dimension 3: patterns of exposure to pornographic material among primary education students

The students’ prior exposure to sexual videos or photographs was investigated, with 43.75% (*n* = 35) responding affirmatively, whereas 52.50% (*n* = 42) denied having had such experience. Three participants did not answer this question. Notably, the number of students who reported understanding the meaning of the term “sexuality” largely coincides with those who reported exposure to sexual content: 57.14% (*n* = 20) of those who reported understanding the term had also been exposed to such material.

In the pre-intervention phase, the most common source mentioned was television series, accounting for 25.58% of the responses (*n* = 11), followed by movies at 13.95% (*n* = 6). Other cited sources included other people and search engines such as Google (9.30% each, *n* = 4). Additionally, isolated mentions were recorded for media such as television, WhatsApp, YouTube, images, magazines, games, or paintings. In one particular case, access to a specific pornographic platform (Pornhub) was reported, although at a low frequency (2.33%).

In the postintervention phase, the series was the main source of exposure, although with a slight percentage decrease to 22.50% (*n* = 9). This was followed by broader mentions such as “videos” (12.50%; *n* = 5) and an increase in the variety of formats identified, including photos, TikTok, YouTube, and movies (7.50% each, *n* = 3). New categories emerged, such as advertisements and reality shows (5% each), and there was a slight increase in the identification of explicit adult platforms such as Pornhub (5%; *n* = 2).

Overall, although series remain the most common source, the intervention revealed greater student awareness of the variety of media through which sexual content can be encountered. Emerging digital platforms and social networks such as TikTok, YouTube, and WhatsApp have gained prominence, potentially reflecting changes in access and consumption patterns. Moreover, the presence of traditional media (television, magazines) decreased or remained low, whereas the number of mentions of explicit content platforms slightly increased. This evolution highlights the need for educational interventions that not only provide clear, age-appropriate information but also foster critical understanding of sexuality and the sexual content that students may be exposed to in different environments. The sources consulted for sexual content are presented in Table 4.

**Table 4:** Sources of pornography exposure.

Source	Pre (Freq.)	Pre (%)	Post (Freq.)	Post (%)
Series	11	25.58%	9	22.50%
Movies	6	13.95%	3	7.50%
Other people	4	9.30%	—	—
Internet (Google)	4	9.30%	2	5.00%
Others/advertisements	3	6.98%	2	5.00%
Television	2	4.65%	—	—
Real life	2	4.65%	1	2.50%
WhatsApp	2	4.65%	1	2.50%
YouTube	2	4.65%	3	7.50%
Paintings	1	2.33%	1	2.50%
Pornhub	1	2.33%	2	5.00%
News	1	2.33%	1	2.50%
Images	1	2.33%	—	—
Magazines	1	2.33%	1	2.50%
Games	1	2.33%	1	2.50%
TikTok	1	2.33%	3	7.50%

*Source*: Authors’ own elaboration.

## 4 Discussion

Comprehensive sexuality education (CSE) aims to equip individuals with the essential tools to manage their health and make informed decisions that promote their wellbeing, thereby contributing to both individual and collective development and transformation (Borazon and Chuang, 2023; UNESCO and WHO, 2023). However, previous studies indicate that knowledge regarding sexuality is insufficient, particularly among school-aged children and adolescents (Myat et al., 2024; Nguyen et al., 2025; Jefatura del Estado, 2020). Therefore, this study focuses on analyzing the short-term changes associated with educational interventions on the level of sexual knowledge among primary school students.

During the classroom intervention, interactive presentations, videos, and activities were employed to facilitate the comprehension of the contents. Nonetheless, the questionnaires indicated that students continued to request a deeper exploration of the topics addressed. Furthermore, many students expressed embarrassment or discomfort when asking questions in public, which limited the expression of their genuine doubts. These findings highlight the need to implement strategies that ensure privacy and anonymity, thereby allowing for a more precise identification of students’ concerns and a better adaptation of the content to their needs (Roldán Restrepo et al., 2021; Corcoran et al., 2020).

Furthermore, the obtained results suggest that a brief sex education intervention yielded relevant changes in the self-reported understanding of basic concepts related to sexuality, particularly regarding the recognition of the term ‘sexuality’ itself and knowledge concerning puberty. These findings are consistent with the evidence synthesized in recent systematic reviews and meta-analyses, which demonstrate that school-based sex education programs significantly contribute to improving knowledge, attitudes, and competencies related to sexual health (Corcoran et al., 2020; Barriuso-Ortega et al., 2024). Nonetheless, this body of evidence also indicates that the most robust and sustained effects are typically observed in comprehensive, longitudinal, and multidimensional programs, whereas brief interventions exhibit a more limited scope, particularly when focusing predominantly on biomedical or preventive contents (Barriuso-Ortega et al., 2024). In this context, although the observed changes must be interpreted with caution given the punctual nature of the intervention and the reliance on self-reported measures, our findings suggest that even short-duration educational initiatives can elicit significant baseline shifts in how students conceptualize and approach sexuality.

Furthermore, following the intervention, a decrease in the discomfort associated with discussing sexual topics was observed, along with an increased perception of normality when addressing these issues. Although this trend did not reach statistical significance, its direction remains clinically and educationally relevant, suggesting that structured sex education can contribute to establishing safer and more open environments for dialogue. Mitigating emotional barriers to discussing sexuality is of paramount importance during childhood, a developmental stage wherein initial attitudes toward these topics can shape future information-seeking behaviors and communication. In this regard, Brouskeli and Sapountzis emphasize the importance of introducing sex education at an early age, promoting not only knowledge but also personal competencies, communication skills, and attitudes that foster the development of a healthy and responsible sexuality (Brouskeli and Sapountzis, 2017).

High-quality sex education constitutes a fundamental pillar for addressing sexuality comprehensively (Jefatura del Estado, 1990). Its efficacy is enhanced when implemented from an early age, coinciding with the onset of secondary sexual characteristics and the development of related psychological processes. CSE stands out as a structured educational approach that integrates relevant contents into school curricula, providing students with tools to comprehend their sexuality and make responsible decisions (UNESCO and WHO, 2023; UNESCO et al., 2025). However, in most European countries, formal sex education initiates between the ages of 7 and 12 (Marquardt, 2022; Alonso-Ruido et al., 2022).

Indeed, in this study, 56.25% of the students at pre-test indicated that they either did not know the meaning of ‘sexuality’ (15.00%) or expressed doubts and uncertainty regarding it (41.25%). At post-test, the proportion of students who affirmed understanding the term increased to 89.86%, whereas uncertainty decreased to 8.70%, and only 1.45% reported not knowing it. This change was statistically significant, demonstrating an association between the study phase and the response (*χ*^2^ de Pearson = 34.89, Fisher’s exact test *p* < 0.001, with a large effect size, Cramér’s *V* = 0.484). Collectively, these findings indicate a significant group-level improvement in self-reported conceptual comprehension following the educational intervention. However, from a methodological standpoint, it is critical to specify that this outcome predominantly reflects self-perceived knowledge—defined as the students’ subjective evaluation of their own comprehension—as opposed to an objective, standardized measure of conceptual literacy.

These results concur with the 2020 European Commission report, which highlights that, although most EU Member States include sex education as a compulsory subject, substantial heterogeneity persists regarding both the age of onset and the contents taught (Velasco-Gijón et al., 2024). Furthermore, a widespread lack of teacher preparedness to address these topics was identified, which could delay students’ access to relevant information (Estrela et al., 2025). In line with these findings, a recent systematic review on initial teacher education regarding sexuality education demonstrated that the preparation of prospective teachers continues to be limited and fragmented, showing substantial variations in curriculum contents, duration, and pedagogical frameworks. The authors underscored that many pre-service teachers exhibit significant knowledge gaps, low self-efficacy, and challenges when addressing sexuality-related topics from a comprehensive, inclusive, and unbiased perspective, potentially compromising the quality and scope of school-based sex education. Additionally, the study emphasized that robust professional training correlates with increased confidence and readiness to implement comprehensive sexuality education (Costello et al., 2022).

Consequently, sex education programs frequently focus primarily on delaying sexual debut and addressing biomedical aspects, such as the prevention of STIs and unplanned pregnancies, whereas affective, emotional, and social components receive limited attention (Lameiras-Fernández et al., 2021). In the multiple-choice item inquiring why it is important to learn about sexuality, the most frequently selected options at pre-test were “to understand changes in puberty” and “to learn about respect and boundaries,” followed by “to know my body.” At post-test, selection rates for the “to know my body” option increased, while those for “I do not know why it is important” decreased; meanwhile, reasons related to pubertal changes and respect/boundaries remained highly selected. Crucially, sex education does not aim to promote sexual activity, but rather to foster responsibility and informed participation in health and emotional development (Sell et al., 2023).

The effective implementation of school-based sex education is intrinsically contingent upon the willingness, competence, and perceived self-efficacy of the teaching staff. Prior evidence maintains that educators face notable structural and curricular constraints, highlighting the restriction of pedagogical time and a manifest absence of specific content within undergraduate and postgraduate curricula (Nguyen et al., 2025; Jefatura del Estado, 1990). These training deficiencies translate into limited knowledge regarding affective-emotional dynamics, which frequently coexists with feelings of insecurity, low self-efficacy, and personal discomfort when addressing sexuality in the classroom (Bruno et al., 2025). Furthermore, recent scientific literature emphasizes that teachers’ attitudes and positioning do not solely depend on their cognitive or conceptual level, but are instead modulated by a complex network of personal, cultural, and institutional factors. Among these, individual beliefs, underlying taboos, social norms, and a chronic fear of punitive reactions from families or academic peers act as critical barriers that restrict the scope of these interventions (Cabrera-Fajardo et al., 2024).

This amalgam of psychopedagogical obstacles fosters a partial or biased implementation of education, favoring predominantly biomedical or preventive approaches to the detriment of the affective, relational, and social dimensions of human development (UNESCO, 2017). Faced with this landscape of insufficient and heterogeneous teacher training, educational institutions bear the deontological responsibility of ensuring a structured curriculum that promotes sex education through approaches based on respect, gender equity, and holistic development (Inchley et al., 2020; Corcoran et al., 2020). In line with this imperative, international bodies such as UNESCO (2021) have urged for an increase in teachers’ formal qualifications, providing them with specific pedagogical tools to address sex education with clarity, sensitivity, and scientific rigor. Thereby, it is imperative to deconstruct institutional taboos and incorporate transversal axes—such as emotional education, a gender perspective, and coeducation—to consolidate safe learning environments and relationships of mutual trust among the school, the student body, and the family community (United Nations, n.d.; Jefatura del Estado, 2020; Cabrera-Fajardo et al., 2024; UNESCO, n.d.).

Although schools serve as the primary agents, the role of the family and the peer group in non-formal sex education should not be underestimated (Widman et al., 2016). In the present study, families were identified as the primary source of information (40%), followed by teachers, healthcare professionals, and friends. The importance of the family is explained by its emotional closeness, trust, and affective availability, which help children and adolescents turn to them when their first sexual concerns arise (Rosengard et al., 2012). Multiple studies have demonstrated that open, non-judgmental communication at home increases the likelihood of children seeking parental guidance, even over that of the school or peers (UNESCO, n.d.; Widman et al., 2016; Rosengard et al., 2012; Pop and Rusu, 2015). However, not all families are equipped to provide scientifically accurate and emotionally healthy information, underscoring the need for school-family collaboration to ensure a comprehensive and contextually relevant sex education (Roldán Restrepo et al., 2021). The study also revealed that healthcare professionals and friends ranked lowest as sources of information due to factors such as limited accessibility, occasional interaction, a lack of specific training, and the circulation of subjective or myth-based information among peers (Akyol et al., 2023; Myat et al., 2024; UNESCO et al., 2025).

Regarding the sources of information, the results of the present study identified the family as the primary reference point when addressing doubts related to sexuality. This finding aligns with recent literature describing the family as a highly valued source due to its emotional closeness and trust. Nonetheless, a critical reading suggests that parental centrality should not necessarily be interpreted as a guarantee of information quality. The same review cautions that parents may experience barriers related to limited knowledge, discomfort, or communication difficulties (Silva et al., 2024). Therefore, the high dependence on the family environment could become a vulnerability factor if the transmitted information is partial, based on beliefs, or reproduces cultural myths. These results reinforce the need to strengthen collaborative models between family and school, avoiding the displacement of educational responsibility exclusively onto one of the two domains.

Conversely, 90.5% of adolescents aged 13–18 reported having consumed pornography (Santa Maria et al., 2015). In the present study, approximately half of the students reported exposure to pornographic content, and many of them demonstrated a lower understanding of sexuality and consent. In Spain, in 2018, the prevalence of online pornography consumption among adolescents aged 12 to 18 reached 70.7% (Sedano Colom et al., 2024). The internet has emerged as the primary space for sexual exploration, followed by television media (Rosengard et al., 2012; Sedano Colom et al., 2024).

Following the intervention, a higher proportion of students reported having viewed pornography. This increase is likely due to a combination of factors. First, it is possible that students felt more comfortable and secure providing honest answers in the post-intervention survey, reflecting a reduction in social desirability bias. Second, the educational sessions may have enhanced the students’ ability to recognize what constitutes sexual content; thus, behaviors or materials that they perhaps did not identify as sexual prior to the intervention could now be accurately reported. These findings underscore the importance of establishing supportive environments and providing clear definitions when assessing sensitive behaviors in adolescents.

Pornography consumption often transmits a phallocentric and violent view of sexuality, which promotes gender inequality, normalizes violence, and centers on male pleasure (Livingstone and Smith, 2014; Rodríguez-Castro et al., 2021). Adolescents who regularly consume pornography are significantly more likely to adopt negative gender attitudes (Stanley et al., 2018). Therefore, it is crucial to reinforce sex education regarding consent, boundaries, and respect, as well as to provide tools to identify and appropriately respond to situations of abuse (Rodríguez-Castro et al., 2021; Stanley et al., 2018).

The present study exhibits methodological limitations that should be taken into account when interpreting these outcomes. First, the single-group pre-test/post-test design, lacking a comparison group, limits the ability to attribute the observed changes exclusively to the intervention, as it precludes ruling out threats to internal validity such as history, maturation, or pre-test effects. Likewise, the anonymous administration of the questionnaires, although ethically justified to guarantee a secure environment and foster honest responses regarding a sensitive topic, prevented the longitudinal matching of responses, thereby restricting the analysis to group-level comparisons between unpaired samples. The generalizability of the findings is also limited due to the use of non-probability sampling, restricted to three educational centers, and the implementation of a single intervention session; consequently, the results must be interpreted as short-term changes associated. Furthermore, the exclusive reliance on self-report measures in child populations may introduce social desirability biases and limit precision when expressing cognitive and emotional aspects related to sexuality. Finally, the predominance of closed-ended questions primarily assesses self-perceived knowledge rather than objective conceptual comprehension, making it difficult to distinguish between actual knowledge acquisition and a greater subjective familiarity with the terms. Consequently, future research should incorporate controlled designs, longitudinal follow-ups, and qualitative measures that allow for a deeper exploration of the conceptual understanding of sexuality.

## 5 Conclusion

The analysis reveals that students possess limited knowledge of basic terms related to sexuality and puberty. Families remain the primary source of information, although increasing internet access and the proliferation of digital content may progressively displace this traditional role. Conversely, there is low confidence in healthcare professionals as reliable sources for addressing these topics, which restricts opportunities for receiving expert guidance.

Health education interventions contribute to the normalization of sexuality-related topics and facilitate more open communication among students. Notably, primary school students express particular concerns related to bodily and psychological changes during adolescence, as well as issues linked to personal boundaries and respect. Some students are unsure how to act or prefer to remain silent in situations of abuse, underscoring the urgent need to strengthen education in these areas.

Finally, the increasing consumption of pornography among youth poses a significant challenge, as both formal and informal comprehensive sexuality education remain insufficient to adequately contextualize and understand this type of content. Therefore, it is imperative to improve and expand comprehensive sexuality education, ensuring that students receive accurate, complete, and developmentally appropriate information.

## Data Availability

The original contributions presented in the study are included in the article/supplementary material, further inquiries can be directed to the corresponding author.
